# Relationship between Urban Green Spaces and Cancer: A Scoping Review

**DOI:** 10.3390/ijerph18041751

**Published:** 2021-02-11

**Authors:** Marion Porcherie, Nyan Linn, Anne Roué Le Gall, Marie-Florence Thomas, Emmanuelle Faure, Stéphane Rican, Jean Simos, Nicola Cantoreggi, Zoé Vaillant, Linda Cambon, Jean-Philippe Regnaux

**Affiliations:** 1 EHESP, French School of Public Health, Av du Pr Léon Bernard, 35043 Rennes CEDEX, France; Anne.Roue-LeGall@ehesp.fr (A.R.L.G.); Marie-Florence.THOMAS@ehesp.fr (M.-F.T.); 2 Arènes UMR CNRS 6051, Université Rennes 1, 140 Bd de la Duchesse Anne, 35700 Rennes, France; nyan.linn@eleve.ehesp.fr; 3 Inserm, EHESP, Irset (Institut de Recherche en Santé, Environnement et Travail)—UMR_S 1085, Univ. Rennes, 35043 Rennes, France; 4 Laboratoire Dynamiques Sociales et Recomposition des Espaces (LADYSS UMR CNRS 7533), Université Paris-Nanterre, 92001 Nanterre, France; efaure@parisnanterre.fr (E.F.); srican@parisnanterre.fr (S.R.); zvaillant@parisnanterre.fr (Z.V.); 5 Campus Biotech—Chemin des Mines 9, Institut de Santé Globale, Université de Genève, 1202 Genève, Switzerland; jean.simos@unige.ch (J.S.); nicola.cantoreggi@unige.ch (N.C.); 6 Equipe MesRI-Inserm U1219, Université de Bordeaux, 146 Rue Léo Saignat, 33000 Bordeaux, France; linda.cambon@u-bordeaux.fr; 7 EpidermE, Univ. Paris Est Creteil, 94010 Creteil, France; jeanphilippe.regnaux@gmail.com

**Keywords:** urban green spaces, cancer, risk factors, contributing factors, scoping review

## Abstract

This scoping study aims to explore the relationships between urban green spaces (UGSs) and the onset, remission and recovery of cancer. We followed the Preferred Reporting Items for Systematic Reviews and Meta-Analyses (PRISMA) extension for scoping reviews (protocol published in 2018). Eligibility criteria for papers were: (1) to be concerned with UGSs, (2) reporting effects of UGSs on cancer-related outcomes including direct or indirect measures, (3) reporting randomized controlled trials, prospective cohort studies, case studies, observational studies, non-comparative studies, (4) in English or French. The search covered primary studies in the published and unpublished (grey) literatures searching by hand and electronic databases (MEDLINE, Green File, Cumulative Index to Nursing and Allied Health Literature and ScienceDirect). Among 1703 records screened by two reviewers independently, 29 were included for qualitative synthesis. We classify the cancers concerned and the effects reported i.e., protective effect, risk or without association. The most investigated cancers are bladder, breast and lung cancer. Our study also identified contributing factors and their mediating effects between UGSs and cancer. Even though the strength of the evidence of the associations between UGSs and cancer is still weak due to the low number of studies and their design, results highlight the wide variety of possible mediating factors between the use of green spaces and cancer occurrence, remission and/or prevention. Knowledge gaps and future research perspectives should be oriented to qualitative research on protective factors with an attention to equity in UGS access and use.

## 1. Introduction

Cancers are, along with cardiovascular diseases, one of the major burdens on the health of the world’s population. The occurrence of cancers is complex and multifactorial and is most often the result of a combination of and interaction between genetic factors and cumulative environmental exposures [1,2,3]. In urban areas, where nearly 55% of the world’s population is now concentrated [4], the search for protective factors linked to the living environment is an important issue. People’s living environments can create health, but the effects can be more or less beneficial depending on the quality of the urban or natural environments being considered [5]. In this context, compounded by climate change and environmental pressure, green spaces could provide an answer because they have many qualities that enable them to have a positive and convincing effect on people’s health [6,7,8,9,10,11]. These protective effects have been demonstrated for different types of health outcome [12]. For example, they provide visitors with a sense of escape from noise and pollution in the city [13] and can thus act as a protective factor for populations against environmental risks such as air or noise pollution or the urban health island effect [14,15,16]. They also induce a strengthening of the immune system and a drop in blood pressure [17]. They act favorably to combat symptoms of anxiety [18], having a positive effect on mental health and reducing stress [19]. They are also places that favor physical activity [20,21] and provide social spaces for individuals and communities [22]. These various protective factors have been highlighted in relation to general mortality and chronic diseases [11]. Among chronic diseases, cardiovascular diseases (CVDs), which are the first cause of death globally [23], and respiratory diseases seem to be particularly reduced by proximity to green spaces [24]. However, these links are less clear for other chronic diseases such as cancer even though they share many of their risk factors with cardiovascular diseases, such as physical activity levels, hypertension, overweight and obesity, or stress and anxiety [25]. The links between green spaces and cancer are multiple and complex [26] but fewer publications have been published in this area than for other chronic diseases. A recent meta-analysis on green spaces and health mentions only four studies on the links between urban spaces and cancer [9]. Another scoping study on the health effects of urban forms on the Canadian population, for example, mentions only one national study involving a link between green spaces and cancer among the 55 studies included [27].

In order to gain a more precise vision of the nature of the research on links between green spaces and cancer and to identify the knowledge needs on the effects of green spaces on cancer, we conducted a scoping review whose protocol was published in 2018 [28]. This review takes place within the framework of the GoveRnance for Equity EnviroNment and Health in the City (GREENH-City) research project [29].

- Review question

Our goal is to explore the relationships between green spaces and cancer, examine the factors affecting the relationship between green spaces and cancer, its pathway (direct or indirect), its direction (risk or protection effect) and examine the factors affecting this relationship. To do so, we will systematically review all the evidence to describe the characteristics of green spaces that have an effect on cancer.

## 2. Materials and Methods

### 2.1. Protocol and Registration

The protocol of our scoping review has been published elsewhere [28]. We followed the Preferred Reporting Items for Systematic Reviews and Meta-Analyses (PRISMA) extension for scoping statement to report our findings (www.equator-network.org/reporting-guidelines/prisma-scr, assessed date: 20 March 2017).

### 2.2. Eligibility Criteria

Studies were included if they met the following eligibility criteria:

- Type of population

The “population” here is urban green spaces. An urban green space refers to any form of plant-covered or plant-containing environment in urban areas. We considered any study related to urban green spaces (UGSs), whether related to their creation, enhancement or maintenance, which can include a large panel of UGSs ranging from small private or public gardens to large public areas such as parks or squares, also including vegetable patches and green façades or roofs [30].

Studies relating to contamination hazards in agricultural soils or cemeteries were not included.

- Types of outcome

Effects of urban green spaces on cancer-related outcomes must be reported, related to the onset of cancer (e.g., carcinogenic data), the remission or the recovery.

- Types of study design

All types of study (randomized controlled trials, prospective cohort studies, case studies, observational studies, non-comparative studies) were eligible for inclusion. Articles such as commentaries, editorials or opinion statements were not considered for inclusion.

### 2.3. Information Sources

The search strategy for this review aims to identify both published and unpublished (grey literature) primary studies and reviews in electronic databases and by manual searching. We searched the following electronic databases for primary studies from database inception up to the search date (last search May 2019). This search was applied to:

- MEDLINE (via PubMed).
- Green File (via EBSCO host).
- Cumulative Index to Nursing and Allied Health Literature (via EBSCO host).
- ScienceDirect.

No restriction date was used. Languages were English and French.

- Other sources

We searched manually in Google and Google Scholar. Journals, reference lists of included studies and previous reviews related to urban green spaces and cancer were hand searched for additional studies. Websites of key organisations involved in addressing and reporting research on green spaces were consulted (World Health Organization (WHO), Agency for Healthcare Research and Quality, National Institute for Health and Care Excellence (NICE), Medical Research National Institute (INSERM), French National Cancer Institute, the Institute of Cancer Research, Commission for Architecture and the Built Environment, national urbanism agencies, etc.). Citation searches of included studies were undertaken using Web of Science (WOS) interface. 

### 2.4. Search Strategy

Keywords and phrases used in the literature search were selected in an attempt to keep both sensitivity and specificity as high as possible. The search strategy was designed and conducted by an Information Specialist (LE) using all identified keywords and index terms across all included databases.

**Figure 1:**
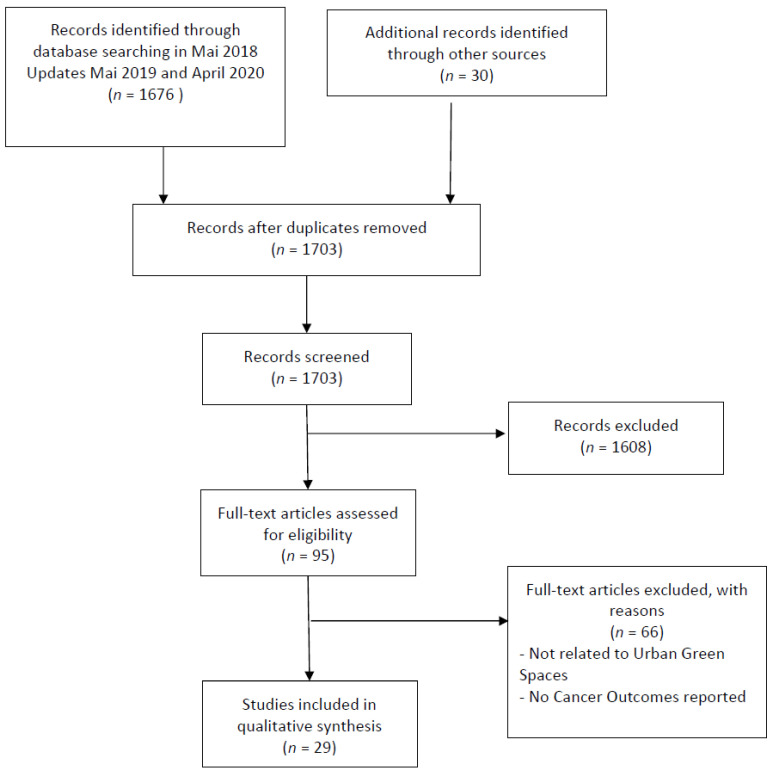
Flow chart of the study selection.

We present a flow diagram of search results and selection of studies in Figure 1.

### 2.5. Selection of Sources of Evidence

We removed duplicate records, then two reviewers (M.P., J.-P.R.) independently screened the titles and abstracts. All articles included after this stage were read in full independently by both reviewers (M.P., J.-P.R.) according to the eligibility criteria. Disagreements during the title/abstract or full text review phases were resolved by consensus.

- Data extraction process

Data were extracted by three authors (M.P., N.L. and J.-P.R), using a data collection form constructed in ExcelTM software (see additional file). To reduce extraction bias, we used a form that had been previously tested and modified by 4 pairs of researchers (S.R.-E.F., J.S.-N.C., A.R.l.G.-M.-F.T., L.C.-M.P.). At the final step, differences between reviewers were resolved by consensus.

- Data items

We extracted the following data from the included studies:

(1) Publication information (title, author and date of publication, journal, country);
(2) Study characteristics (design, population of interest, aims and objectives of the studies);
(3) Green space characteristics: type of green space, method used to characterize green space, factor related to green space that may have an effect on cancer;
(4) Cancer outcomes: type of cancer measures reported (ex: reported number of skin cancer, number of breast cancer survival …)
(5) Effects on cancer: types of cancer involved, relation to exposure (direct and/or indirect) and direction of findings for each study (risk, protection, no association);
(6) Other links/relationships (issues that might be of interest addressed by the study).

### 2.6. Quality Appraisal of Included Studies

The methodological quality of the selected studies was assessed during data extraction. We used the appropriate critical appraisal sheets available from https://casp-uk.net/casp-tools-checklists/ (accessed date: 20 March 2017) to assess the quality of RCTs, observational studies (OS), systematic reviews (SR), case-control studies (CC), cohort studies (CS) and qualitative design studies (QDS). We used the critical appraisal tool AXIS developed by Downes et al. (2016) [31] for cross-sectional studies, as well as the Critical Appraisal Checklist for Quasi-Experimental Studies (QES) developed by the Joanna Briggs Institute (JBI) [32].

Critical appraisal sheets were then used to qualitatively assess the methodological rigour of each study. The appraisal sheets contained 27 criteria for cross-sectional studies and OS (AXIS tool), 18 criteria for RCTs, 20 for CS, 16 for SR, CC and QDS, and 11 for QES. We considered a study to be of good quality (++) when all criteria were met, of fair quality (+) when most of the criteria were met and of poor quality (–) when most of the criteria were not met. Assessments were made by a first reviewer (NL) then all studies were cross-checked by a second reviewer (MP or JPR) for discrepancies.

### 2.7. Analysis

Data extracted from the selected studies were coded by content analysis, grouped by categories and reported in a data extraction table. We examined the data-extraction chart corresponding to each study included in the review. We used R v3.4.2 (the R Foundation Statistical Computing, Vienna, Austria) to compute frequencies and percentages.

### 2.8. Narrative Synthesis

We performed a narrative synthesis. To determine the influence of urban green spaces on cancer, we classified and pooled the findings using frequency and synthesis tables. We performed analysis by cancer types and direction of effects (risk, protection, no association). To identify the direction of the relationship between urban green spaces and cancer, we considered that the relationship was “a risk effect” when the risk of cancer or the presence of carcinogenic substances was statistically increased, a “protective effect” when it statistically decreased, “no effect” when the difference was not statistically significant. We also pooled data of exposure or contributing factors for cancer in all the included studies.

Three members (M.P., N.L., J.-P.R.) classified each study according to the type of relationship (direct or indirect) between exposure to UGS and cancer. The relationship was classified as direct when the main purpose of the study was to investigate the association between green space and cancer while using statistical methods and analyses to estimate a quantity/area/amount of green space. In any other situation, the type of relationship was classified as indirect.

We also analysed the identified contributing factors that might mediate effects between green spaces and cancer in each study. These factors are presented in Section 3.5.

## 3. Results

### 3.1. Results of the Literature Search

The first literature search was in December 2017. It was updated in May 2019 and again in April 2020.

- December 2017

We identified 1580 citations after removing duplicates and excluded 1462 studies after screening titles and abstracts. In total, 60 full-text reports were selected for evaluation. After assessing all records, we included 20 studies.

- Update in May 2019

We searched the electronic databases listed in the methods section for study reports published between December 2017 and May 2019. The search resulted in 35 new records, of which 11 were assessed in detail and six were selected.

- Update in April 2020

We searched the listed electronic databases for reports published between June 2019 and April 2020. The search identified 47 new records all assessed in detail and three were selected.

A flow chart shows the overall search process in Figure 1.

### 3.2. Study Characteristics of Included Studies

The main characteristics of the included studies are summarized in Table 1. The full data extracted from each study are presented in the Supplementary Materials (Table S1). We included a total of 29 reports, which were published between 2008 and 2020. Most of the studies were conducted in China (*n* = 10, 34%), the United States (*n* = 8, 28%) and the United Kingdom (*n* = 7, 18%). One study combined data from multiple countries [33]. Of the 29 studies, most (*n* = 19, 66%) were cross sectional studies, four (14%) were cohort studies, two (7%) were case control studies, one (3%) was a randomized controlled trial, one (3%) was a qualitative study and one (3%) was a systematic review.

Study population and samples were different across studies: 20 studies (69%) were carried out at city level, six (21%) at a nationwide level, two (7%) at a regional level, and one (3%) was unspecified. Table 1 shows that sample size of the target population varied widely from one study to another, ranging from 14 people to 43 million people. The age of the samples varied across studies. Some included only adults and people aged over 65 (*n* = 9, 31%), while others included both adults and children (*n* = 10, 34%) or adults and teenagers (*n* = 3, 10%). Others included only children (*n* = 2, 7%). Some studies did not provide information on the age of people in the sample (*n* = 5, 17%). The majority of studies did not specify the gender of people within the samples (*n* = 19, 66%). Four studies focused exclusively on women (14%) and two only men (7%). Samples of substances concentration were collected by half of the studies. Eleven (38%) analyzed concentration in soil, three in dust (10%) or one in water (7%) in green space locations.

- Green Space Characteristics

Different types of green space were considered in the included studies. Most of them were parks including playgrounds or recreational areas. Eleven studies (38%) used spatial variables to estimate land coverage or distance from residential address. Green space was estimated using vegetation density index (*n* = 4), census unit (*n* = 4), meshblock classification (*n* = 1) or proportion of land cover (*n* = 2). The size of green spaces varied greatly, from individual spaces measuring from 0.25 km^2^ to 1 km^2^, to the percentage of green spaces over a total city area. Different levels of green space coverage between city were found and ranged from 19% to 69%.

- Cancer related outcomes

The types of cancer reported in the included studies can be found in Table 1 and Table 2. Cancer (mainly carcinogenic risk) was used as an outcome in 11 studies (38%). Lung cancer was the most studied (*n* = 5, 17%). One study [34] investigated the impact on several cancers (lung, larynx, urinary bladder). Fourteen studies (48%) assessed the carcinogenic risk of substances (soil, air, dust or water). Three studies did not report any finding on cancer outcomes but examined the impacts of green spaces on cancer survivors [35,36] or the prevention of skin cancer [37]. One of the 29 study focused on recovering from cancer [35].

### 3.3. Quality of Included Studies

The evaluation of the quality of the studies is reported in Table 1. After evaluation, six (21%) studies received a high quality score, 22 (76%) fair quality and one (3%) was rated low quality.

### 3.4. Effects on Cancer Found in Studies

The 29 studies were classified according to the types of UGS effects on cancer outcomes observed in each study. In accordance with the methodology described above, we analysed the direction of effects and defined three categories: risk effect, protective effect, and no significant association. These effects by type of cancer are reported in Table 2.

- Bladder cancer risk

One study [34] found no statistical association between bladder cancer risk and green space. We considered the evidence as indirect

- Breast cancer

Three studies examined the relationship between urban green spaces and breast cancer. One study [38] reported a higher statistically significant risk of mortality due to breast cancer for women who live in neighborhoods with parks compared to those without parks. Because there was no statistically significant difference in the adjusted models, we classified the evidence as indirect. Two studies reported a protective effect. O’Callaghan-Gordo et al. (2018) [39] found direct evidence of a reduced risk of breast cancer for participants who lived with urban green spaces around their residence (300 m) compared to those who did not. English, Wilson, and Keller-Olaman (2008) [35] interviewed 14 women with breast cancer and identified indirect evidence that natural landscapes including parks can help in the recovery of cancer survivors by inspiring feelings of calmness through a sense of connection with nature.

- Skin cancer risk

We found two studies that explored the risk of skin cancer associated with urban green spaces. One study [40] provided direct evidence of an increased risk. One study [37] found no statistical association.

- Lung cancer

Five studies have examined the association between green space and lung cancer. Based on direct [41,42,43] and indirect [33,44] evidence, there was no statistically significant relationship between green space availability and lung cancer mortality.

- Larynx cancer risk

One study [34] reported indirect evidence showing no significant statistical association between incidence of cancer and Danish gardeners who were exposed to pesticides.

- Cancer mortality

James et al. (2016) [44] conducted a study including 108,630 women to test the link of exposure of greenness and mortality. Women living in greenest areas around their home (250 m) had a lower rate of all-cause mortality. Based on direct evidence, they found a protective effect of UGSs which were consistent for an area up to 1250 m (although weaker).

- Carcinogenic risk

Among 15 studies exploring the relationships between green space and carcinogenic risks, three found an increased risk [45,46,47], one reported a protective effect [48] and 11 studies [49,50,51,52,53,54,55,56,57,58,59] found no statistical association. All the evidence was indirect.

- Prostate cancer risk

One study [54] assessed whether living in the proximity of greener areas was related to prostate cancer incidence in a city population. There was direct protective evidence showing that men living in greener areas had a lower statistical risk of prostate cancer.

### 3.5. Correlates/Effect Modifiers of Associate with Urban Green Spaces and Cancer

**Figure 2:**
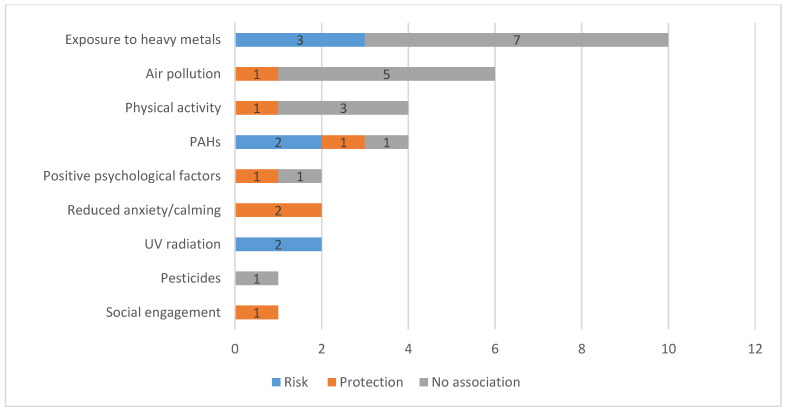
Contributors to the relation between green space and cancer investigated in the studies included (*n* = 29).

Figure 2 shows the contributing factors and their mediating effects in the relation between urban green spaces and cancer investigated in the studies included.

Based on the 29 selected studies, nine main contributing factors involved in the relation between UGS and cancer were identified during the extraction of data (Figure 2). Exposure to heavy metals was the most tested factor (*n* = 10, 34%). Of the 10 studies, three (30%) found an increased risk effect [46,56,57]; seven (70%) observed no statistical association [49,51,52,53,55,58,59]. Air pollution was considered as a contributing factor in seven studies (24%), of which five (71%) found no association [39,41,54,60,61], one (14%) reported a protective effect [44]. Physical activity was tested in four studies (14%). Among these four studies, one found a protective effect [46] and three of them reported no association [38,39,61]. The other contributing factors identified among all the studies were polycyclic aromatic hydrocarbons (PAHs) (*n* = 4, 14%), positive psychological factors (*n* = 2, 7%), reduced anxiety (*n* = 2, 7%), ultraviolet (UV) radiation (*n* = 2, 7%), pesticides (*n* = 1, 3%) and social engagement (*n* = 1, 3%).

Despite a global high quality of studies included, we have too few study giving a direct link to made any conclusion of the strength of evidence neither for the relation between UGS and cancer not for the contributing factors (air pollution, physical activity, social engagement …) in the onset, remission or recovery from cancer. Nevertheless, our studies provide an overview of current issues in this area of research, the key points of which are discussed below.

## 4. Discussion

This study provided a comprehensive systematic review of the literature on the links between urban green spaces and cancer.

### 4.1. Principal Findings: A Diversity of Exposure Modalities

The results of the 29 studies about the links between cancer and urban green spaces show that half were considered non-specific carcinogenic risks (Table 2). The other studies enabled us to identify the types of cancer most investigated in relation to urban green space, namely lung, breast and skin cancers. The results highlight a diversity of exposure modes resulting from a variety of risk situations, through air contact with the skin or airways, or by ingestion of substances present in the air, soil or water. From this point of view, the studies focus more on risk situations than on causal effects linked to the very nature of green spaces, such as their surface, aesthetic characteristics, or plant species. When they do investigate causality, such studies tend to look at protective factors related to the use of green spaces during a period of cancer remission or relapse. For the whole body of work, the effects studied are mostly of an indirect nature. In fact, only 8 studies (28%) look at direct causal links between green spaces and cancer, i.e., measuring a link between a surface/presence of UGSs and a risk of cancer [39,40,41,43,44,54,61].

### 4.2. Comparison with Previous Studies: Identification of Mediation Factors

Our scoping review confirms the variability of results from previous reviews on the links between green space and health [9,62,63]. Nevertheless, by focusing more specifically on cancer, it allows us to go further in identifying contributing factors as mediators of the relationship between urban green spaces and cancer. We identified nine mediators of various natures, which can be grouped into two types according to whether they induce a protective or risk effect for cancer. The first type of mediation factor, which has a protective effect, acts on individual characteristics such as mental well-being by lowering anxiety levels or strengthening positive psychological factors physical well-being by promoting physical activity, and social well-being by encouraging social involvement. The latter category of mediation factors, which have a rather deleterious effect, are external exposure factors present in green and blue spaces. These include heavy metals, chemicals such as PAHs and pesticides, UV exposure and air pollution. Many of these latter mediators lack conclusive results to show their deleterious effects on cancer; this is particularly the case for studies on heavy metals. Nevertheless, these substances present a potential carcinogenic risk [60] and, therefore, cannot be ignored. All of these mediating factors can potentially play a role in the relationship between green spaces and the occurrence, remission, relapse or protection of cancer. Studies looking at the links between green spaces and cancer should, therefore, consider as many of these different mediators as possible. These mediators are highly dependent on the inherent characteristics of the UGS, which is defined in particular by its physical, social and landscape features.

**Figure 3:**
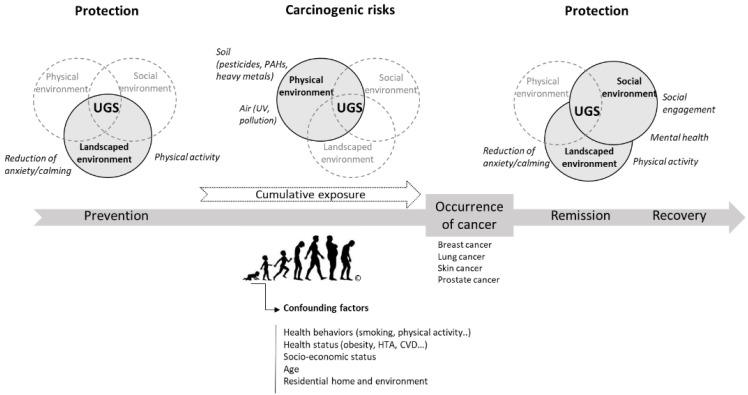
Identification of major components of UGSs (physical, social and landscaped environment) and their mediating factors *(text in italics)* related to the risk or protection of cancer prevention, occurrence, remission and recovery from the 10 studies (on 29) demonstrating a link between UGSs and cancer. Doted lines indicate the component on which research should be developed. Picture: © Robert Adrian Hillman.

Figure 3 synthetizes data extracted from the 10 studies (see Table 2) showing an association between UGSs and cancer risk or protection.

### 4.3. Transferability: The Role of Contextual Variables

The diversity and low number of reviewed studies question the transferability of the results of this scoping exercise [64], especially since the results were produced in local contexts [65,66,67]. The analysis highlights three types of contextual factors: the nature of green spaces (recreational area, urban park, pocket park, etc.), the urban forms in which they are embedded (in a hyper-urbanized area, in proximity to main roads, in a small city…) and the factors related to the population (age, socio-economic status, health behaviors…).

Indeed, the carcinogenic risk can be more or less attenuated and sometimes less important in green spaces depending on the mediator, which may depend strongly on the nature of the urban green space (its size, its location area, its context in the urban fabric, its age, etc.). These data can also vary greatly from one country to another (historic parks in European cities or new installations in industrial areas, etc.). Research covers countries with very different forms of urbanization. For example, China, Australia, Canada and Spain are some of the countries where present urban patterns differ significantly. The level of integration of green spaces into the urban fabric is also a factor to take into account. UGSs can, for example, be more or less closed off regarding a neighboring road which is a source of air pollution and noise, and may be easily accessible (e.g., close to the city center) or further afield. All of these contextual factors may have an influence on the quality and quantity of mediation factors that can be found in green spaces. 

Moreover, one of the major difficulties in highlighting the links between cancer and green spaces lies in the multifactorial causality of cancers. It is obvious that in urban areas, as elsewhere, any interpretation of results must take into account numerous confounding factors that make it more difficult to demonstrate a direct link experimentally. Only some of the quantitative studies include adjustment factors in the analysis models. When carcinogenic risks are calculated, the models are most often age-adjusted with a difference between children and adults. Some studies also include behavioral factors such as tobacco consumption or social characteristics, such as the level of deprivation of individuals or of the geographical area under consideration. These choices depend on the model but also on the type of cancer examined. Respiratory cancers will typically be more sensitive to data on smoking, which can be linked to socio-economic status. According to Dzhambov et al. (2020) [68], these third factors which act on the relationship between the mediator and the green space can modulate the effects. Our study shows that too few studies take into account contextual variables, particularly deprivation factors in risk situations. In addition, very few studies develop lifelong approaches (apart from taking into account medical history) when cancer occurs in a situation of cumulative exposure at all ages. 

### 4.4. Quality of Evidence Reliability of this Scoping Review

These results stem from the analysis of 28 studies. For the most part, they are epidemiological or observational and their designs do not allow for the determination of a causal link. Evidence of direct links remains difficult to establish, or would require a different methodological approach: a large-scale cohort study to take into account the occurrence of cancer, under consideration of adjustment factors and a large set of mediators. Moreover, few studies show a direct effect, which would represent stronger evidence for the link between UGSs and cancer. Furthermore, we found a certain heterogeneity between studies with regard to cancer-related variables. However, this remains inherent to the multiplicity of cancers that may be involved and to the measurement methods used. Additionally, a certain heterogeneity also appears in the way in which UGSs are assessed, which can potentially concern a wide variety of spaces with very different health impacts (large grass-covered park, small concrete-covered playground …). The different nature of these UGSs might be coupled with more or less “green” covering (canopies, trees…).

### 4.5. Strengths and Limitations of this Scoping Review

This work has limitations. First, the databases were chosen to cover both medical and environmental journals to represent the two poles of our research. Precautions were taken to ensure double or even triple reading and consensus conferences for all stages of decision-making. Nevertheless, the final selection of the articles rests on some subjectivity on the part of the reviewers. Several people in the research group looking for consensus, although subjective, assessed the thematic rankings. Two researchers (double check), carried out extractions, although not systematic. Once again, the extracted data were selected if the two researchers reached consensus. The broadness of the selection criteria led to discussions during the inclusion phase. Then we excluded certain studies that might have been of interest in understanding the links between green spaces and cancer, such as studies focusing on preventive behaviors (e.g., wearing sunglasses or a sunscreen in parks).

Other limitations lie in the definition of green spaces that we adopted by limiting ourselves to urban spaces, excluding treeless recreation areas and private gardens in urban areas. However, they can also have an impact and modulate the occurrence of cancer.

### 4.6. Knowledge Gap and Future Research Perspectives

A large proportion of the selected studies focus on chemicals present in green spaces through dust, in the soil or in bathing or recreational water accessible in these spaces, that may be risk factors for cancer. In opposition, very few studies adopt a salutogenic perspective considering UGSs as a determinant of health for the individuals who use them [10] and whose characteristics in terms of layout, plant species [69], configuration or accessibility may have an influence on the individual and social behaviors of populations [70,71].

Our results show that there are few qualitative studies investigating the psychological factors that play a major role in supporting people with cancer. However, we know that green spaces and their various components have a considerable influence on the mental health of the populations that use them [72,73,74]. Researches should target psychological factors among populations in remission or undergoing cancer treatment. This would help to better distinguish between the benefits to be expected from the use of a green space and what is likely to have the greatest effect: its available surface area, the biodiversity, the practice of physical activity, the vision of the green, the presence of other people, etc. This role of public green spaces among people in remission from cancer, based on the model of therapeutic gardens that are being provided in healthcare establishments [75,76], should also become a topic of scientific investigation. These were not included in the scope of our review, but this other part of the literature deserves to be investigated in the light of recent publications on the benefits of contact with natural spaces for health [77] and regarding cancer in particular.

Some recent studies show a direct link between the production of natural killer cells by the immune system and contact with nature, thus suggesting a beneficial effect on cancer [78,79,80]. It would be interesting to carry out this type of study in urban green spaces that are equitably accessible to the greatest number of people. These studies would also prove whether, despite exposure to co-factors such as poor air quality, urban green spaces are still beneficial for populations in remission from cancer. This risk-benefit approach, more commonly used in studies on environment-dependent cancers, would deserve (1) to be more systematic in any study of the impact of urban green spaces on health and (2) to cover a wider range of co-factors belonging to the three main dimensions of a green space (social, environmental and landscape). In this way, more evidence data would be available to guide planning choices for urban green spaces in order to make the most of their ecosystem, human, social and economic functions [63].

The effects of green spaces are also dependent on (i.) the number of UGSs available, (ii.) distance to the nearest UGSs, (iii.) frequency of park visits, and (iv.) the view on to a park from home [81]. It should be determined which of these components affect the carcinogenic risk in particular. Cohort studies on populations exposed on a daily basis to professionals working in green spaces would be interesting. Indeed, apart from the known risks linked to pesticides, these professionals are also exposed to all the other co-factors we have mentioned. Such monitoring would also make it possible to study protective factors. Finally, our results strongly question the lack of consideration for vulnerable populations. Research focusing specifically on vulnerable populations (related to age, gender, health, socio-economics status, etc.) should be developed because it is these populations that are likely to benefit most from green spaces [82,83,84,85,86].

## 5. Conclusions

Our study shows the paucity of research specifically oriented towards interactions between urban green spaces and cancer. It also highlights the wide span of possible mediating factors between green spaces and cancer occurrence/remission/prevention. Nevertheless, it shows varied results in terms of risk and protection of green spaces according to the mediating factors to which the population may be exposed. In view of this, the precautionary principle should be applied to the risk factors.

## Figures and Tables

**Table 1:** General characteristics of the included studies (*n* = 29).

Reference Number	Authors, Date	Country	Study Design	Study Population	Setting of Exposure	Cancer Outcomes	Quality Assessment
[33]	Gascon et al. (2015)	UK (n = 4), USA (n = 5), Canada (n = 1), Lithuania (n = 1), New Zealand (n = 1)	Systematic review and meta-analysis	12 articles (no gender specific)	Green or blue space exposure was assigned based on location of residence	Lung cancer mortality	++
[34]	Kenborg et al. (2017)	Denmark	Cohort	3124 (>35 years, men)	Greenhouses, nursery gardens, or public parks, gardens and cemeteries	Diagnoses of smoking-related cancers from national registers	++
[35]	English et al. (2008)	Canada	Qualitative	14 (adult and over 65, women)	Parks, open fields, and street trees, views of trees from a window, potted plants, and backyard gardens.	Cancer recovering	++
[36]	Nakau et al. (2013)	Japan	Quasi experimental	22 (adult and over 65, no gender specific)	The Japan World Exposition ’70 Commemorative Park	Cancer patients	-
[37]	Hedges et al. (2010)	UK	Qualitative	100 (adult, both sexes)	Two London parks	Skin cancer risk	+
[38]	Keegan et al. (2014)	USA	Cohort	4345 (adult and over 65, women)	Parks included beaches, recreation areas, and parks	Survival after diagnosis of breast cancer	+
[39]	O’Callaghan-Gordo et al. (2018)	Spain	Case–control	1129 breast cancer cases (adult and over 65, women)	Around each participant’s address of residence: Public green areas, gardens, zoos, parks, forests, tree canopy.	Risk of breast cancer	++
[40]	Astell-Burt et al. (2014)	Australia	Cross-sectional	267 000 (adult, over 65, no gender specific)	Public green spaces near residential area within 1km area	The prevalence of melanoma and non-melanoma skin cancer	+
[41]	Bixby et al. (2015)	UK	Cross-sectional	149 369 deaths (teenager, adult, both sexes)	% of Green spaces coverage of the 50 largest cities in England	Observed number of deaths from lung cancer	+
[42]	Richardson et al. (2010)	New Zealand	Cohort	Over 1.5 million (teenager, adult, both sexes)	Natural areas (e.g., parks, beaches, and fields) but excluded aquatic areas (e.g., lakes and the sea)	Lung cancer mortality	+
[43]	Richardson et al. (2012)	USA	Cross-sectional	43 million (both sexes)	Green space coverage in each city	Lung cancer mortality	+
[44]	James et al. (2016)	USA	Cohort	108 630 ( adult and over 65, women)	Vegetation around each participant’s home address	cancer-specific mortality in the Nurses’ Health Study	++
[45]	Ke et al. (2017)	China	Cross-sectional	Over 10 million (children and adult, no gender specific)	Public parks and playgrounds	Total cancer risk (TCR)	+
[46]	Guney et al. (2010)	Turkey	Cross-sectional	10 million (children, no gender specific)	Seventeen playgrounds (10 with treated wood, 4 with metal and 3 with plastic structures), 4 parks and 3 picnic areas	probabilistic carcinogenic risk for As uptake	+
[47]	Zhang et al. (2016)	China	Cross-sectional	21.6 million of residents	Four differents types of urban green spaces	Carcinogenic risk	+
[48]	Mihankhah et al. (2020)	Iran	Cross-sectional	13 million (children and adult)	Urban parks	Carcinogenic risk	+
[49]	Brtnicky et al. (2018)	Czech Republic	Cross-sectional	Over 370 000	Park	Carcinogenic risk	+
[50]	Chen et al. (2013)	China	Cross-sectional	31 million	Lake in urban green spaces (blue component)	Carcinogenic risk	+
[51]	Frimpong et al. (2019)	Ghana	Cross-sectional	57.6 % of the total population of Ghana	Public parks with passive recreation areas	Carcinogenic risk	+
[52]	Gu et al. (2017)	China	Cross-sectional	Over 12 million (children and adult)	Parks	Carcinogenic risk	+
[53]	Hiller et al. (2017)	Slovak Republic	Cross-sectional	466 000 (children, no gender specific)	Playgrounds in Public kindergartens and urban parks soils	non-carcinogenic and carcinogenic health risks	+
[54]	Demoury et al. (2017)	Canada	Case–control	3 927 (adult and over 65, men)	Greenness measured within home buffers of 150 m, 300 m, 500 m and 1000 m	Prostate cancer risk	++
[55]	Lu et al. (2019)	China	Cross-sectional	7.5 million	Land-use nammed as green lands	Carcinogenic risk	+
[56]	Wang et al. (2016)	China	Cross-sectional	8.19 million (children and adult, no gender specific)	Parks in the center of Nanjing	Carcinogenic risk	+
[57]	Xia et al. (2011)	China	Cross-sectional	21.54 million of residents	Large public green space and classical garden	Carcinogenic risk	+
[58]	Yang et al. (2018)	China	Cross-sectional	NA (children and adult)	Typical recreational garden	Carcinogenic risk	+
[59]	Zhao et al. (2017)	China	Cross-sectional	3.55 million	Urban parks	Carcinogenic risk	+
[60]	Li et al. (2017)	China	Cross-sectional	Urban population of about 15.7 million	Park areas	Carcinogenic risks	+
[61]	Richardson et al. (2010)	UK	Cross-sectional	2,9 million of people (teenager and adult, both sexes)	Green spaces ranged from transport verges and neighbourhood greens, to parks, playing fields and woodlands.	Lung cancer mortality	+

NA: Not available. Quality assessment: study of good quality (++), fair quality (+) and poor quality (–).

**Table 2:** The effects with direction observed in the included studies on cancer outcomes. The link was considered to be direct when the investigation of the relationship between green space and cancer was stated as an objective of the study, an outcome measure of cancer rate was used, the design and the statistical analysis were suitable to establish a relationship. In other cases or where it was not clearly described, the evidence was considered to be indirect.

Effects	Risk	Protection	No Association
Bladder cancer risk			**Indirect**: [34]
Breast cancer risk	**Indirect**: [34]	**Direct**: [39] **Indirect**: [35]	
Skin cancer risk	**Direct**: [40]		**Indirect**: [37]
Lung cancer risk			**Direct**: [41,42,43,61] **Indirect**: [33,34]
Larynx cancer risk			**Indirect**: [34]
Non specific Cancer mortality		**Direct**: [44]	
Non-specific carcinogenic risk	**Indirect**: [45,46,47]	**Indirect**: [48]	**Indirect**: [49,50,51,52,53,55,56,57,58,59,60]
Prostate cancer risk		**Direct**: [54]	

## Supplementary Materials

The following are available online at https://www.mdpi.com/1660-4601/18/4/1751/s1, Table S1: Data extraction.
